# Improving the Aromatic Profile of Plant-Based Meat Alternatives: Effect of Myoglobin Addition on Volatiles

**DOI:** 10.3390/foods11131985

**Published:** 2022-07-05

**Authors:** Jolien Devaere, Ann De Winne, Lore Dewulf, Ilse Fraeye, Irena Šoljić, Elsa Lauwers, Andy de Jong, Hermes Sanctorum

**Affiliations:** 1 KU Leuven Technology Campus Ghent, Gebroeders De Smetstraat 1, 9000 Ghent, Belgium; jolien.devaere@kuleuven.be (J.D.); ann.dewinne@kuleuven.be (A.D.W.); lore.dewulf@kuleuven.be (L.D.); ilse.fraeye@kuleuven.be (I.F.); 2 Paleo b.v., 12 Rue des Pr. Jeener et Brachet, 6041 Gosselies, Belgium; irena.soljic@paleo-taste.com (I.Š.); elsa.lauwers@paleo-taste.com (E.L.); 3 Paleo b.v., Meilrijk 98, 3290 Diest, Belgium; andy.dejong@paleo-taste.com

**Keywords:** myoglobin, plant-based meat alternatives, HS-SPME-GC-MS, volatiles, PCA-analysis, aroma, Maillard reaction, lipid oxidation, aldehydes, pyrazines

## Abstract

Market demand for palatable plant-based meat alternatives is on the rise. One of the challenges is formulating products with sensorial characteristics similar to conventional meat. In this study, the effect of myoglobin on the aromatic profile of plant-based meat alternatives was assessed. Plant-based burgers were made with soy-textured protein, supplemented with three levels of myoglobin (0, 0.5 and 1.0%, the latter two mimicking endogenous myoglobin levels in meat), and grilled for 12 min at 250 °C. To evaluate the aromatic profile of the compounds, raw and grilled samples were subjected to headspace solid-phase microextraction (HS-SPME) followed by gas chromatography-mass spectrometry (GC-MS). Principal component analysis (PCA) analysis was then performed to visualize the interaction between grilling and myoglobin addition, and the effect exerted on the resulting aromatic profile. Myoglobin significantly affected several classes of volatile compounds, either by itself or in conjunction with grilling. A notable increase in aldehydes and a decrease in hydrocarbons were noted after adding myoglobin. As expected, an increase in pyrazines was observed after grilling. The results suggest myoglobin positively influences the aromatic profile of plant-based meat alternatives, contributing to a profile closer to the one of conventional meat.

## 1. Introduction

Meat consumption has a long history in human evolution, likely going back to the earliest known human-like ancestor living 5–7 million years ago [1]. Environmental but also health and animal welfare concerns regarding conventional meat production and consumption are the main drivers for developing meat alternatives [2,3,4,5]. To appeal to the largest consumer segment that is not committed to vegetarian or vegan diets, the food industry is placing extra emphasis on introducing meat alternatives to consumer markets. One of the aims is formulating plant-based products with similar sensorial (texture, color, flavor) [6,7] and nutritional characteristics to conventional meat [7].

Flavor and aroma are complex attributes of meat palatability. Cooking meat involves a series of reactions resulting in the development of various volatile compounds. These include Maillard reactions, lipid oxidation, interactions between Maillard reaction products and lipid oxidation products, and thiamine degradation [8]. Hundreds of volatile compounds result from these reactions, e.g., aldehydes, alcohols, carboxylic acids, ketones, pyrazines and esters. Saturated and unsaturated aldehydes, especially those containing 6–10 atoms of carbon, are a major contributor to the volatile profile and flavor development of cooked meat [9,10]. Roast flavors in foods are usually associated with the presence of heterocyclic compounds such as pyrazines, thiazoles and oxazoles. In well-done grilled meat, pyrazines are reported to be the major class of volatiles [11].

The inherent flavor and aromas of a meat product can be influenced by its lipid content [12] and pH [13], the diet, age and gender of the animal [14], and the presence of myoglobin [15]. Myoglobin is a globular heme protein found in muscles, capable of reversible oxygen binding via a heme-bound iron atom [16]. It is important for the sensory quality of meat and has been associated with a serum-like taste and metallic mouthfeel of beef [17]. The close relationship of heme proteins (hemoglobin and myoglobin) with lipid oxidation during cooking has been extensively studied [18,19,20]. Myoglobin has long been proposed to contribute to aroma development by catalyzing lipid oxidation reactions [21,22]. Lipid oxidation products in turn promote myoglobin oxidation and alter its stability [23].

Upon cooking, myoglobin unfolds, exposing the heme cofactor. The cofactor then catalyzes a series of reactions that transform amino acids, nucleotides, vitamins and sugars in the meat into a variety of flavor and aroma compounds characteristic of the complex aromatic profile of cooked meat. Consistent with the important role of heme iron in meat aroma, a plant heme protein, is now commercially used to optimize flavor in ground beef analogues intended to be cooked [24]. This heme protein, called Leghemoglobin, is originally found in the root nodules of leguminous plants.

Some published studies assess the safety, toxicity, and allergy potential of plant heme-protein in plant-based meat alternatives [25,26]. However, to our best knowledge, there is no publicly available data on the influence of myoglobin, the muscle heme protein, on the aromatic profile of meat alternatives. In the present proof-of-concept study, we used gas chromatography-mass spectrometry (GC-MS) to assess the effect of myoglobin on the formation of volatile compounds. The perspective here is to evaluate the potential of fermentation-derived, animal-free myoglobin as functional ingredient for plant-based meat alternatives. The information presented here could therefore be of value to plant-based food manufacturers interested in ameliorating the sensory properties of their products and formulating a product closer to conventional meat.

## 2. Materials and Methods

### 2.1. Preparation of Meat Alternatives (Raw and Grilled) with Addition of Commercial Myoglobin

Plant-based burger alternatives were produced using 57.5% (m/m) of reverse osmosis (RO) water, 25% (m/m) textured soy protein (TSP) (Fuji Oil, Ghent, Belgium), 15% (m/m) sunflower oil (Vandemoortele, Ghent, Belgium), 1% (m/m) methylcellulose (50D, Snick Euroingredients, Ruddervoorde-Oostkamp, Belgium) and 1.5% (m/m) table salt, with the on top addition of either 0%, 0.5% or 1% (m/m) of commercial bovine Mb (Tebu-bio, Boechout, Belgium). First, TSP was hydrated in water (45%) for 30 min at 15 °C. Next, methylcellulose, salt and sunflower oil were added, after which the mixture was grinded using a plate with 4 mm openings (meat mincer, Minerva Omega Group, Bologna, Italy). Then, the Mb was dissolved in water (12.5%) and mixed with the batter. Raw hamburgers (30 g, Ø 6 cm) were shaped manually and baked for 12 min at 250 °C in an oven (Rational Climaplus Combi CPC 61, Paal, Belgium).

### 2.2. HS-SPME-GC-MS Analysis

The volatile compounds of burger samples were isolated by means of headspace solid-phase microextraction (HS-SPME) using a Gerstel MPS2 autosampler fitted with a 50/30 µm Divinylbenzene/Carboxen/Polydimethylsiloxane (DVB/Carboxen/PDMS) SPME fiber (Supelco, Bellefonte, PA, USA). Prior to analysis, the SPME fiber was conditioned for 30 min at 270 °C, according to the manufacturer’s instructions.

Three grams (± 0.1%) of sample material was transferred into 20 mL glass headspace vials, sealed with aluminum crimp caps lined with PTFE/silicone septa and stored in a cooled tray at 4 °C until analysis. Samples were equilibrated at 45 °C for 20 min with intermittent agitation at 250 rpm (5 s on/2 s off). During the last five minutes of incubation, the SPME fiber was conditioned at 270 °C and then immediately exposed to the vial headspace for 40 min at 45 °C to extract the headspace volatiles.

Extracted compounds were subsequently separated and analyzed using an Agilent 7890A/5975C GC-MS system equipped with an Agilent HP-1ms capillary column (30 m × 0.25 mm × 0.25 µm). The SPME fiber was desorbed for 5 min in the GC inlet at 250 °C using a 0.75 mm ID HS-SPME liner (Supelco, Bellefonte, PA, USA), in splitless mode. An initial oven temperature of 35 °C was held for 5 min and increased at 4 °C/min to 215 °C, followed by a second ramp of 7 °C/min to a final oven temperature of 250 °C, which was held for 5 min. Helium (99.9999%) was used as a carrier gas at a constant flow rate of 1.2 mL/min.

The MS detector was operated in Electron impact ionization (EI) mode with an ionization energy of 70 eV. The source and quadrupole temperatures were set to 230 and 150 °C, respectively. Mass ranges were scanned between 40 and 250 m/z.

### 2.3. Data Analysis

Volatile organic compounds (VOC) were tentatively identified by matching mass spectra with MS data libraries (NIST08, WILEY275) and by comparing their linear retention indices (LRI) with the literature. LRI are calculated using Van Den Dool and Kratz’s equation for temperature programmed GC conditions, in which *t_x_* is the retention time of compound “*x*”, and *t_n_* and *t_n+_*_1_ are the retention times of n-alkanes (C_6_–C_16_) with carbon number “*n*” eluting before and after compound “*x*”:

(1) $${\left(LRI\right)}_{x}=100n+100\times \frac{t_{x}-t_{n}}{t_{n+1}-t_{n}}$$

Identified VOCs were classified according to their chemical nature: organic acids, alcohols, aldehydes (saturated, unsaturated, branched and cyclic), hydrocarbons, ketones, phenols, pyrazines and others. The peak areas of individual compounds as well as the total peak areas of each class of compounds are reported as area units (AU) × 10³ (mean ± standard deviation) of HS-SPME-GC-MS analysis performed in triplicate.

The effect of Mb addition (% Mb), grilling (G) and their interaction (% Mb × G) on individual volatiles or groups of compounds was statistically analyzed through two-way ANOVA using IBM SPSS 27. In case of a significant interaction, the interaction term was further interpreted using one-way ANOVA followed by a post-hoc Tukey’s honestly significant difference (HSD) test. A significance level of *p* < 0.05 was employed.

Principal component analysis (PCA) was carried out using The Unscrambler X (v. 10.5.1) on a multivariate dataset containing peak areas of all identified compounds in each analyzed burger. Data were pre-processed by mean-centering and scaling to unit variance prior to analysis. The first two principal components (PCs) were considered to visualize the interaction between grilling and myoglobin enrichment of meat alternatives, and the resulting aromatic profile.

## 3. Results and Discussion

### 3.1. Volatile Profile

A total of 40 volatile compounds were identified in the analyzed samples (Table 1), of which 3 appeared to be exclusive to grilled samples: furfural, furfuryl alcohol and pyrrole. The volatile profile of raw meat alternatives (RMA), without addition of myoglobin (Mb), consists mainly of hydrocarbons and 2-pentylfuran, which account for 34.0 ± 6.7% and 29.7 ± 2.2% of the total peak area respectively, followed by alcohols (13.7%) and unsaturated aldehydes (12.0%). Grilling the plant-based meat alternatives (GMA) is associated with a significant decrease in alcohol and cyclic aldehyde content, in favor of the formation of branched aldehydes, ketones, phenols and most notably pyrazines. Despite this shift in volatile fractions, the grilling process does not significantly affect the total peak area of volatiles present in RMA compared to GMA. However, addition of myoglobin (Mb) leads to significant (*p* < 0.05) formation of various odor-active volatile compounds. Statistical analysis further indicates a significant interaction between effects of Mb addition and grilling (%Mb × G) on the total peak area, suggesting that a synergetic relationship exists between both factors.

A total of 11 saturated and unsaturated aldehydes are identified in the plant-based meat alternatives, supplemented with Mb. Most of these aldehydes are typically associated with (auto-)oxidation of unsaturated fatty acids in food matrices [27]. Due to their low odor thresholds, aldehydes are known to greatly impact the aroma of meat and meat products. Of all classes of chemical compounds, the peak area of saturated aldehydes displays the most pronounced increase with rising Mb concentrations, making them the dominant fraction of the volatile profile in Mb-enriched samples. In RMA + 1.0%Mb and GMA + 1.0%Mb, they respectively make up 36.9 ± 1.0% and 30.2 ± 1.3% of the total peak area. Hexanal is the most abundant aldehyde in the analyzed samples. In high concentrations, it imparts an unpleasant rancid odor, but at low levels it is characterized by a pleasant, grassy aroma and generally contributes to a desired aroma in meat products [28,29]. RMA contains high initial levels of hexanal (10.6 ± 1.3% of the total peak area), which may originate from the soy protein used to prepare the burgers. This aldehyde is reported as the predominant volatile compound in soybeans, accounting for 40.9% of the total volatile profile of the ingredient [30]. Grilling found to reduce the amount of hexanal in GMA. Hexanal can also be formed as an oxidation product of linoleic acid and is often considered as an excellent indicator of lipid oxidation in meats and meat products rich in n-6 polyunsaturated fatty acids [31]. Addition of Mb in both RMA + Mb and GMA + Mb leads to considerable increases in hexanal content which greatly exceeds the decrease caused by the grilling process in GMA. Additionally, the highest peak areas for hexanal are found in GMA + Mb. This suggests that the addition of Mb has a significant (*p* < 0.01) influence on the degree of lipid oxidation in RMA + Mb and GMA + Mb, which is further amplified by the heat of the grilling treatment. Further research is required to determine whether these hexanal concentrations exceed rancid odor thresholds or remain within a desirable range. Among other identified aldehydes, heptanal and nonanal (saturated aldehydes derived from oleic acid), and unsaturated aldehydes exhibit similar, increasing tendencies in relation to Mb concentration. Branched aldehydes (2- and 3-methylbutanal), on the other hand, originate from proteolysis and degradation of amino acids [27], and exhibit an opposite behavior, whereby they decrease as the Mb content increases in both raw and grilled samples.

The volatile profile of meat products and the analyzed plant-based meat alternatives appears to differ fundamentally in terms of hydrocarbon content, which represents a substantial portion of the total peak area in RMA (34.0 ± 6.7%) and GMA (32.9 ± 2.1%). Hexane is the most abundant hydrocarbon observed in all analyzed samples. While it is not naturally present in soybeans [30,32], it may be a residue from lipid extraction using hexane as a solvent during the production of soy protein [33]. HS-SPME-GC-MS analysis of raw ingredients, performed under identical conditions to those of the sample analysis, indicated that hexane was the most abundant volatile compound in the TSP used to prepare the plant-based burgers (data not shown). In meat matrices, hydrocarbon compounds are generally reported in low levels and are not considered to contribute significantly to meat-like aromas [34,35,36,37,38]. They are considered to originate from the thermal oxidative decomposition of lipids, catalyzed by heme compounds such as hemoglobin and myoglobin [6]. Research data on aroma development during the heat treatment of meat products is limited and not conclusive regarding hydrocarbon contents. Contrary to the understanding of how they are formed, short-chain carbohydrates (<C14) are mostly reported to degrade or to be unaffected during the cooking of beef and pork [7,10]. In GMA grilling does not affect the hydrocarbon content either. On the other hand, addition of Mb is found to reduce the amount of hydrocarbon compounds (except octane) to 13.7 ± 1.1% and 6.9 ± 0.7% of the total peak in RMA + 1.0%Mb and GMA + 1.0%Mb, respectively. Further research is required to determine whether excess hydrocarbons pose challenges with regards to flavor in meat alternatives, but if so, the addition of Mb offers potential to reduce hydrocarbon levels and better mimic the natural volatile profile of meat.

All six pyrazine compounds identified in the analyzed samples have previously been reported in beef, pork, chicken and mutton [34]. Pyrazines are derived from Maillard reactions and their presence in meat matrices is mainly associated with roasted aromas [39,40,41]. Small amounts of pyrazines are initially present in RMA, and do not vary with Mb addition. They are likely formed during the extrusion-cooking process in the production of TSP. Temperatures at which soy protein are heated during extrusion generally range from 120 to 180 °C, at which point pyrazines can be formed via the Maillard reaction [42,43,44]. HS-SPME-GC-MS analysis of raw materials (data not shown) confirmed the presence of all six pyrazines in TSP, and similar findings have previously been reported in the literature [45]. Grilling is expected to increase the pyrazine content in plant-based meat alternatives considerably, but Mb supplementation in GMA is found to further stimulate pyrazine formation significantly (*p* < 0.05). Interactions are known to occur between products derived from lipid-oxidation and intermediates of the Maillard reaction [46,47]. The pyrazine fraction of the total volatile profile increases from 11.9 ± 3.0% in GMA to 16.1 ± 2.3% in GMA + 1.0%Mb. Other Maillard-related compounds, such as furfural and furfuryl alcohol [48], are found to exhibit similar patterns. These compounds are not detected in RMA and display a rising trend in function of Mb concentration in combination with grilling. Mb addition increases pyrazine formation in grilled samples, possibly by enhancing the Maillard browning reaction, thereby improving the desired roasted aromatic profile in plant-based meat alternatives.

Beyond aldehydes, hydrocarbons and pyrazines, other aromatic compounds found in meat alternatives include organic acids, alcohols, ketones, phenols and others (pyrrole, 2-pentylfuran and maltol). The amounts of acidic compounds display large fluctuations between the different repetitions, but no significant changes are observed as a function of the grill treatment or the addition of Mb. All remaining volatile compounds are generally found to increase with rising Mb concentrations, except for 2-heptanone, 2-methoxy-4-methylphenol and pyrrole.

### 3.2. Multivariate Analysis

**Figure 1:**
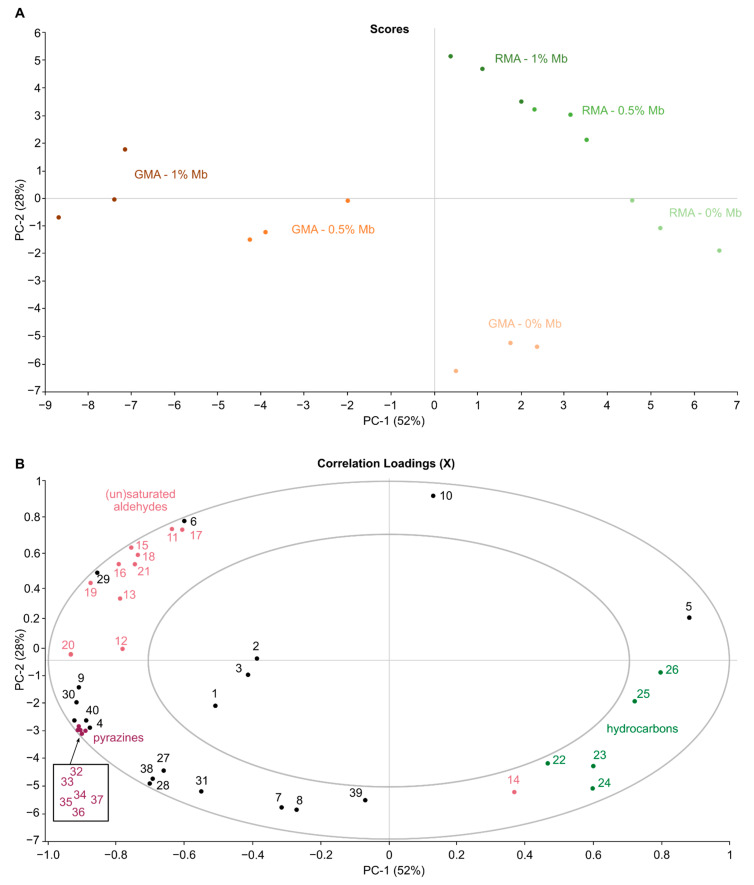
PCA scores (**A**) and correlation loadings (**B**) plots of PC1 and PC2 for volatile compounds in raw and grilled plant-based burgers enriched with varying concentrations of myoglobin. Volatiles (loadings) are denoted by their numbers in Table 1 [Acids (1–3), Aldehydes (7–21), Hydrocarbons (22–27), Pyrazines (32–37)].

The results from Table 1 revealed that the total peak areas of the different meat alternatives were significantly influenced by both addition of Mb and grilling as well as their interaction. PCA is conducted to visualize the relationship between plant-based meat alternatives in terms of their volatile profile (Figure 1). The score plot (Figure 1A) shows that the first two principal components (PC) explained 79.8% of the total variability. All VOCs (n = 40) are shown in the plane of the first 2 PC (PC1 and PC2); the circles indicate if variables are reconstructed at the 50% (inner circle) and 100% (outer circle) of the total explained variance (Figure 1B).

The first PC, accounting for 52% of variance, separates GMA + Mb samples from GMA, RMA and RMA + Mb. Additional contribution of PC2, explaining 28% of variance, drives scores upwards as a function of increasing Mb concentration and down as a result of grilling. In the resulting score plot, raw and roasted samples are clearly separated by a diagonal line. Grilling causes a strong increase in pyrazine content, which is characterised by a shift towards the lower left. Additionally, the samples migrate along the separation line towards the upper right corner as a function of Mb addition. Evidence for a significant interaction between Mb addition and grill treatment is apparent from the fact that the direction of RMA and RMA + Mb shifts to their corresponding GMA and GMA + Mb scores are not parallel, and from the greater distance between GMA and GMA + Mb clusters, compared to RMA and RMA + Mb. The addition of Mb is necessary for aroma precursor formation whereas grilling is mainly responsible for transforming these precursors into volatile components. While most volatile components clearly play a role, acids (1–3) appear to be less important. GMA + Mb were mainly described by saturated (11–13) and unsaturated aldehydes (15–21) and pyrazines (32–37). As explained above, RMA are characterized by mainly (branched) hydrocarbons (22–26), 1-hexanol (5) and decanal (14).

## 4. Conclusions

Formulating meat alternatives that are attractive for a large consumer segment is essential to meet the ever-growing global protein demand. The results from the present study indicate that supplementing meat alternatives with myoglobin has the potential to enhance the volatile profile in a desirable way.

## 5. Patents

Data included in this manuscript are part of an international patent application No. PCT/EP2021/087884.

## Figures and Tables

**Table 1:** Peak areas of volatiles components (average AU × 10³ ± standard deviation × 10³, n = 3) identified in raw and grilled meat alternatives enriched with varying concentrations of commercial myoglobin (Mb). Significance of main effects of % Mb, grilling (G) and their interaction (% Mb × G) following two-way ANOVA analysis are indicated (NS = not significant). In case the interaction term was found to have a significant effect, Tukey’s HSD test was conducted instead of two-way ANOVA of the main effects (- = not analyzed), in which case values within the same row not sharing the same subscript were found significantly different between treatments at *p* < 0.05.

N°	Compound	Unknown LRI ^1^	Reference LRI ^1^	Raw Meat Alternatives (RMA)			Grilled Meat Alternatives (GMA)			2-Way ANOVA Significance (Alpha = 0.05)		
				RMA	RMA + 0.5%Mb	RMA + 1.0%Mb	GMA	GMA + 0.5%Mb	GMA + 1.0%Mb	% Mb	G	% Mb × G
	**Acids**			**204.67 ± 37.24**	**163.53 ± 34.15**	**304.27 ± 50.84**	**235.46 ± 160.10**	**1093.37 ± 1199.55**	**568.79 ± 377.98**	**NS**	**NS**	**NS**
1	Acetic acid	625	625	179.22 ± 33.03	118.01 ± 49.49	208.00 ± 46.91	212.77 ± 172.16	309.64 ± 101.23	263.82 ± 173.09	NS	NS	NS
2	Octanoic acid	1162	1169	5.52 ± 4.95	17.15 ± 8.81	34.27 ± 21.24	8.47 ± 7.44	101.15 ± 149.02	42.01 ± 17.96	NS	NS	NS
3	Nonanoic acid	1258	1268	19.93 ± 8.77	28.36 ± 10.44	62.00 ± 50.16	14.23 ± 12.45	682.58 ± 1015.61	262.95 ± 214.72	NS	NS	NS
	**Alcohols**			**3685.55 ± 524.65**	**4684.63 ± 591.67**	**5586.83 ± 163.30**	**1545.67 ± 228.85**	**3364.99 ± 174.73**	**4244.33 ± 318.35**	**0.000**	**0.000**	**NS**
4	Furfuryl alcohol	837	826	n.d. ^a^	n.d. ^a^	n.d. ^a^	68.35 ± 36.08 ^a,b^	89.64 ± 35.55 ^b,c^	155.34 ± 36.25 ^c^	-	-	0.030
5	1-Hexanol	855	850	2976.13 ± 416.67	2089.81 ± 442.57	1950.16 ± 274.80	1116.23 ± 209.27	727.07 ± 96.73	386.26 ± 46.11	0.001	0.000	NS
6	1-Octen-3-ol	966	983	709.42 ± 109.36	2594.82 ± 310.85	3636.68 ± 399.18	361.09 ± 24.91	2548.28 ± 164.25	3702.73 ± 301.48	0.000	NS	NS
	**Branched Aldehydes**			**5.15 ± 8.91 ^a^**	**14.28 ± 24.73 ^a^**	**9.76 ± 16.90 ^a^**	**506.23 ± 21.30 ^c^**	**192.92 ± 48.22 ^b^**	**199.87 ± 41.69 ^b^**	-	-	**0.000**
7	3-Methylbutanal	625	622	5.15 ± 8.91 ^a^	9.66 ± 16.73 ^a^	5.73 ± 9.93 ^a^	143.27 ± 15.05 ^c^	66.47 ± 10.18 ^b^	63.01 ± 8.83 ^b^	-	-	0.000
8	2-Methylbutanal	635	627	n.d. ^a^	4.62 ± 8.00 ^a^	4.03 ± 6.98 ^a^	362.96 ± 9.83 ^c^	126.45 ± 38.42 ^b^	136.86 ± 34.70 ^b^	-	-	0.000
	**Cyclic Aldehydes**			**1146.19 ± 107.85**	**1742.39 ± 299.87**	**2177.20 ± 300.31**	**699.86 ± 105.60**	**1167.18 ± 107.89**	**1368.04 ± 25.49**	**0.000**	**0.000**	**NS**
9	Furfural	799	802	n.d. ^a^	n.d. ^a^	n.d. ^a^	16.15 ± 27.97 ^a^	106.27 ± 52.19 ^b^	148.46 ± 35.45 ^b^	-	-	0.005
10	Benzaldehyde	922	921	1146.19 ± 107.85	1742.39 ± 299.87	2177.20 ± 300.31	683.71 ± 78.49	1060.91 ± 68.71	1219.58 ± 60.78	0.000	0.000	NS
	**Saturated Aldehydes**			**3193.57 ± 343.90 ^a^**	**10,662.48 ± 1011.73 ^b^**	**13,330.93 ± 481.47 ^c^**	**1556.37 ± 173.13 ^a^**	**11,275.58 ± 921.93 ^b^**	**14,951.23 ± 41.23 ^c^**	-	-	**0.002**
11	Hexanal	775	771	2834.01 ± 321.13 ^b^	10,340.95 ± 916.87 ^c^	12,848.83 ± 420.92 ^d^	1201.11 ± 176.28 ^a^	10,800.63 ± 911.84 ^c^	14,297.20 ± 11.47 ^d^	-	-	0.002
12	Heptanal	877	874	151.18 ± 16.64 ^a,b^	114.66 ± 63.14 ^a^	165.32 ± 14.29 ^a,b^	124.27 ± 7.53 ^a^	213.31 ± 7.45 ^b,c^	253.48 ± 9.39 ^c^	-	-	0.004
13	Nonanal	1081	1083	198.36 ± 24.19	206.87 ± 44.04	316.78 ± 63.56	212.23 ± 8.77	261.64 ± 23.14	400.55 ± 26.92	0.000	0.012	NS
14	Decanal	1183	1203	10.02 ± 9.15	n.d.	n.d.	18.77 ± 2.25	n.d.	n.d.	0.000	NS	NS
	**Unsaturated Aldehydes**			**64.91 ± 11.69 ^a^**	**1713.19 ± 333.87 ^b^**	**3028.08 ± 716.24 ^c^**	**111.56 ± 3.34 ^a^**	**2412.52 ± 257.87 ^b,c^**	**4291.35 ± 375.27 ^d^**	-	-	**0.046**
15	2-Heptenal	928	951	n.d. ^a^	910.59 ± 130.13 ^b^	1482.92 ± 264.78 ^c^	n.d. ^a^	1135.85 ± 136.92 ^b,c^	1984.58 ± 147.95 ^d^	-	-	0.036
16	2-Octenal	1030	1061	34.27 ± 1.89 ^a^	310.70 ± 64.42 ^b^	563.49 ± 167.38 ^c^	29.13 ± 8.54 ^a^	396.22 ± 62.10 ^b,c^	908.21 ± 79.95 ^d^	-	-	0.010
17	(E,E-)-2,4-Nonadienal	1182	1199	8.68 ± 7.53	373.74 ± 113.42	719.18 ± 210.16	n.d.	338.64 ± 40.71	735.01 ± 87.60	0.000	NS	NS
18	2-Decenal	1233	1256	0.85 ± 1.47	23.09 ± 8.46	55.54 ± 19.43	1.31 ± 2.27	30.08 ± 3.60	74.49 ± 8.48	0.000	NS	NS
19	(E,Z-)-2,4-Decadienal	1263	1268	n.d. ^a^	19.97 ± 7.63 ^a,b^	48.12 ± 15.24 ^b^	n.d. ^a,b^	56.37 ± 4.00 ^c^	81.44 ± 9.77 ^c^	-	-	0.004
20	(E,E-)-2,4-Decadienal	1283	1288	18.65 ± 4.85 ^a^	66.21 ± 26.82 ^a,b^	133.86 ± 34.23 ^b^	79.56 ± 2.31 ^a,b^	441.46 ± 44.42 ^c^	472.97 ± 57.88 ^c^	-	-	0.000
21	2-Undecenal	1334	1350	2.47 ± 2.61	8.89 ± 4.20	24.96 ± 10.36	1.56 ± 1.37	13.90 ± 2.04	34.65 ± 3.66	0.000	NS	NS
	**Alkanes**			**9537.27 ± 3932.97**	**4757.54 ± 1085.68**	**4946.03 ± 605.80**	**9807.61 ± 521.13**	**4839.94 ± 633.10**	**3430.74 ± 447.39**	**0.000**	**NS**	**NS**
22	2-Methylpentane	<600	553	577.36 ± 431.74	262.44 ± 111.86	290.99 ± 30.33	602.01 ± 74.63	321.95 ± 91.95	232.19 ± 28.72	0.020	NS	NS
23	3-Methylpentane	<600	590	1091.26 ± 558.45	521.94 ± 142.57	562.94 ± 91.92	1107.55 ± 86.87	531.33 ± 112.32	375.19 ± 63.76	0.001	NS	NS
24	Hexane	600	600	5502.04 ± 1770.46	2556.70 ± 468.23	2619.76 ± 338.67	6235.53 ± 306.44	2686.46 ± 304.45	1835.29 ± 209.84	0.000	NS	NS
25	Methylcyclopentane	618	618	1125.23 ± 673.07	687.57 ± 146.88	658.26 ± 54.99	807.21 ± 93.13	443.10 ± 75.67	338.51 ± 78.14	0.032	NS	NS
26	Cyclohexane	647	647	1159.83 ± 485.84	693.62 ± 196.39	760.50 ± 92.52	712.66 ± 63.45	395.53 ± 44.83	322.60 ± 47.96	0.013	0.003	NS
27	Octane	800	800	81.56 ± 20.14 ^a^	35.28 ± 30.63 ^a^	53.59 ± 6.93 ^a^	342.65 ± 6.78 ^b^	461.57 ± 23.92 ^c^	326.96 ± 30.81 ^b^	-	-	0.000
	**Ketones**			**625.06 ± 72.65**	**715.46 ± 134.18**	**796.79 ± 18.06**	**2708.99 ± 53.96**	**2488.64 ± 514.06**	**3340.68 ± 361.13**	**0.021**	**0.000**	**NS**
28	2-Heptanone	868	870	582.86 ± 66.40	458.72 ± 83.76	434.12 ± 59.12	2674.78 ± 53.78	2088.24 ± 518.36	2723.19 ± 399.77	NS	0.000	NS
29	2,3-Octanedione	963	966	42.21 ± 6.30 ^a^	256.74 ± 53.32 ^b^	362.67 ± 41.37 ^c^	34.21 ± 0.97 ^a^	400.41 ± 22.52 ^c^	617.49 ± 39.43 ^d^	-	-	0.000
	**Phenols**			**12.62 ± 3.70**	**n.d.**	**0.19 ± 0.33**	**185.83 ± 60.22**	**169.72 ± 31.93**	**310.78 ± 105.83**	**NS**	**0.000**	**NS**
30	Guaiacol	1056	1052	0.57 ± 0.99 ^a^	n.d. ^a^	0.19 ± 0.33 ^a^	52.99 ± 32.95 ^a,b^	94.46 ± 29.05 ^b^	205.96 ± 46.20 ^c^	-	-	0.001
31	2-Methoxy-4-vinylphenol	1070	1060	12.05 ± 2.83	n.d.	n.d.	132.85 ± 50.37	75.26 ± 11.17	104.82 ± 61.59	NS	0.000	NS
	**Pyrazines**			**300.22 ± 28.26 ^a^**	**270.90 ± 60.00 ^a^**	**285.85 ± 34.30 ^a^**	**3557.19 ± 948.44 ^b^**	**4717.95 ± 1057.69 ^b^**	**7969.41 ± 1309.12 ^c^**	-	-	**0.001**
32	Methylpyrazine	794	796	21.31 ± 20.28 ^a^	51.44 ± 20.80 ^a,b^	36.19 ± 31.53 ^a^	495.34 ± 123.11 ^b,c^	802.87 ± 226.49 ^c^	1266.36 ± 305.34 ^d^	-	-	0.006
33	2,5-Dimethylpyrazine	882	884	101.22 ± 4.32 ^a^	79.74 ± 17.63 ^a^	91.92 ± 2.71 ^a^	1264.96 ± 366.65 ^b^	1649.87 ± 388.37 ^b^	2938.08 ± 510.88 ^c^	-	-	0.001
34	2-Ethyl-6-methylpyrazine	971	970	24.39 ± 4.90 ^a^	20.31 ± 2.23 ^a^	23.61 ± 1.16 ^a^	321.32 ± 100.02 ^b^	410.41 ± 112.05 ^b^	710.70 ± 145.65 ^c^	-	-	0.005
35	2-Ethyl-5-methylpyrazine	973	973	57.26 ± 10.19 ^a^	41.63 ± 7.28 ^a^	51.90 ± 7.34 ^a^	647.55 ± 134.84 ^b^	850.09 ± 167.10 ^b^	1390.69 ± 158.40 ^c^	-	-	0.000
36	2,5-Dimethyl-3-ethyl-pyrazine	1053	1053	89.18 ± 11.76 ^a^	77.79 ± 13.32 ^a^	79.78 ± 2.08 ^a^	701.87 ± 196.78 ^b^	853.54 ± 179.08 ^b^	1392.55 ± 198.91 ^c^	-	-	0.002
37	2-Methyl-3,5-diethyl-pyrazine	1132	1138	6.87 ± 4.10 ^a^	n.d. ^a^	2.46 ± 4.26 ^a^	126.15 ± 28.06 ^b^	151.17 ± 27.13 ^b^	271.03 ± 57.79 ^c^	-	-	0.002
	**Others**			**8461.00 ± 1206.46 ^b^**	**5849.08 ± 417.59 ^a^**	**5719.21 ± 224.49 ^a^**	**8937.64 ± 28.79 ^b^**	**8661.31 ± 152.26 ^b^**	**8847.58 ± 969.56 ^b^**	-	-	**0.009**
38	Pyrrole	727	731	n.d.	n.d.	n.d.	280.33 ± 67.51	232.82 ± 117.28	270.24 ± 71.31	NS	0.000	NS
39	2-Pentylfuran	978	994	8001.63 ± 1252.79 ^b^	5354.32 ± 177.22 ^a^	5136.56 ± 258.42 ^a^	7706.66 ± 314.55 ^b^	7365.33 ± 194.02 ^b^	7196.63 ± 798.75 ^b^	-	-	0.011
40	Maltol	1276	1272	459.38 ± 88.24	494.76 ± 242.44	582.65 ± 118.56	950.65 ± 222.92	1063.15 ± 22.85	1380.71 ± 107.83	0.024	0.000	NS
	**Total volatiles**			**27,236.22 ± 6183.06 ^a^**	**30,573.47 ± 3231.45 ^a,b^**	**36,185.15 ± 2222.29 ^b,c^**	**29,852.42 ± 480.67 ^a,b^**	**40,384.13 ± 1512.33 ^c^**	**49,522.78 ± 1936.45 ^d^**	-	-	**0.035**

^1^ Linear retention index (LRI) based on 30 m HP-1ms column. Reference values obtained by comparison with sources from the literature in the NIST Chemistry WebBook (https://webbook.nist.gov/chemistry, accessed on 20 May 2022) using similar columns and similar temperature programs.

## Data Availability

Data is contained within the article.
